# A survey of resistance mutations to reverse transcriptase inhibitors (RTIs) among HIV-1 patients in northeast of Iran

**DOI:** 10.22099/mbrc.2024.48729.1895

**Published:** 2024

**Authors:** Zahra Mazaheri, Sahar Tahaghoghi-Hajghorbani, Kazem Baesi, Kiarash Ghazvini, Saeid Amel-Jamehdar, Masoud Youssefi

**Affiliations:** 1 Department of Microbiology and Virology, School of Medicine, Mashhad University of Medical Sciences, Mashhad, Iran; 2 Immunogenetic and Cell Culture Department, Immunology Research Center, School of Medicine, Mashhad University of Medical Sciences, Mashhad, Iran; 3 Hepatitis and AIDS Department, Pasteur Institute of Iran, Tehran, Iran; 4 Antimicrobial Resistance Research Center, Mashhad University of Medical Sciences, Mashhad, Iran

**Keywords:** HIV, Reverse Transcriptase Inhibitors, drug resistance, Iran

## Abstract

The use of a combination of three-drug regimen has improved HIV-1 infected patients' life span and quality; however the emergence of drug-resistant strains remains a main problem. Reverse transcriptase inhibitors (RTIs) consist of a main part of highly active anti-retroviral therapy (HAART) regimen. The present study aimed to investigate resistant mutations to RTI drugs in both treatment naïve and under treatment HIV patients in Mashhad city, north-eastern Iran. RNA was extracted from sera of 22 treatment naïve and 22 under treatment patients. The mean age of under treated and treatment naive groups were 38.5±6.7 and 40.8±7.9 respectively. cDNA was synthesized and amplified with Nested PCR assay targeting specific sequences of RT gene. The PCR products were sent for sequencing. Bidirectional sequencing results were analysed using HIV drug resistance database supplied by Stanford University (HIV Drug Resistance Database, https://hivdb.stanford.edu). Among under treatment patients 10 out of 22 (45%) had at least one high-level resistance mutation which was higher than high level resistance mutation rate among treatment naive cases (P<0.01). Detected resistance mutations were as follows: K101E, K103N, K103E, V106M, V108I, E138A, V179T, Y181C, M184V, Y188L, Y188H, Y188F, G190A, L210W, T215F, T215Y, K219Q, and P225H. A high level of resistance mutations to RT inhibitors was observed that causes drug resistance especially against lamivudine (3TC). Such mutations should be considered as probable responsible for therapeutic failure. Serial surveillance studies of circulating drug resistance mutations are recommended.

## Introduction

The devastating alarm of acquired immunodeficiency syndrome (AIDS) pandemic [1]. as ‘the plaque of the century’[2]. has drawn significant global health attention and concern [3]. Until this time, there has been a notable progress regarding to the Human Immunodeficiency Virus type 1 (HIV-1) pathogenesis and disease management research [4], nevertheless the consequences of HIV-1 infection are still a challenge facing humanity [2]. It seems that HIV-1 induced infection has been a unique disease in terms of rapid antiviral development. There are several types of drugs currently used in a combination anti-retroviral therapy (ART). The classes of drugs are categorized into protease inhibitors (PIs), integrase inhibitors, reverse transcriptase inhibitors (RTIs) and fusion inhibitors [5]. However soon new challenge of drug side effects [6] and resistant mutant variants appeared [7, 8]. 

Among anti-retroviral drugs, RTIs are the main component of the combination regimens currently used. These drugs inhibit RT function which mediates the main phenomenon of retroviruses: RNA to ds DNA synthesis. The drugs are categorized into two distinct groups: i) nucleoside/nucleotide analogue reverse transcriptase inhibitors (NRTIs) which act as chain terminators when are inserted in growing DNA strand, [9, 10] and, ii) non-nucleoside reverse transcriptase inhibitors (NNRTIs) which bind and change the conformation of RT enzyme, thereby inhibit the enzyme activity [11]. Main NRTIs consist of abacavir (ABC, Ziagen), didanosine (ddI, Videx), Emtricitabine (FTC, Emtriva), Lamivudine (3TC, Epivir), Stavudine (d4T, Zerit), Tenofovir (TDF, Viread), Zalcitabine (ddC), Zidovudine (ZDV. While most common prescribed NNRTIs are NVP or nevirapine and DLV or delavirdin [12].

The NNRTI class has a very high chemical diversity because this class consists of more than 50 families of molecules. Unlike chemical heterogeneity, NNRTIs all bind to RT in the same pocket region. In particular, efavirenz has the highest antiviral activity among NNRTIs. First-generation NNRTIs include: delaviridine (Rescriptor), Efavirenz (Sustiva), and Nevirapine (Viramune). Since the lipophilic pocket is mainly made up of three sites of the significant amino acid sequence of RT, all, except one of the NNRTI resistance mutations, are found in the same three regions (amino acids 98–108, 178–190, and 225-238) [13]. 

Resistance to NRTIs is created by selecting strains of HIV-1 that have several mutations in the RT gene of the virus. Two distinct mechanisms of resistance to NRTIs have been described: i) nucleoside analogue mutations (NAMs) such as Y115F, F116Y, V118I, Q151M, and M184V which confer affinity reduction of RT to the drug; and ii) Thymidine analogues mutations (TAMs) such as L210W, T215Y, and K219Q / E which are mutations selected by the thymidine analogs AZT and d4T. They reduce NRTI susceptibility through primer unblocking mechanism [13, 14]. Due to the high mutation rate of RT gene, resistance to NNRTIs occurs more rapidly; however, more effective drug regimens can be achieved by combining different classes such as RT and PIs combination [13, 15-17]. 

In Iran, the disease was first discovered in a haemophilia patient (1986) [18, 19] and, over the years, the spread of the HIV-1 induced infection tended to increase slightly. In 1996, the first outbreak was reported from a prison among IV drug abusers [18, 20]. The patients indicated for anti-retroviral therapy are treated based on national protocol, however, screening for drug mutations is not routinely performed and regimen is usually replaced by a physician upon treatment failure. Therefore, the present study was performed to screen circulating drug resistance mutation among two groups of the patient with and without a history of treatment. 

## MATERIALS AND METHODS

**Study groups:** The study was performed in Mashhad city, north east of Iran. HIV-1 positive patients were selected from the patients with no specific racial characteristics who referred to Mashhad central health centre. Totally 44 patients were included among whom 26 were men. The participants were divided in two groups: 22 treatment naïve and 22 under treatment patients. The group under treatment received standard national therapy regimen including Zidovudine (NRTI), Lamivudine (NRTI), and Efavirenz (NNRTI) for at least one year. The mean age of under treated and treatment naïve groups were 38.5±6.7 and 40.8±7.9 respectively. Inclusion criteria was middle-aged (30-55 years) patients with complete data files who agreed to participate in the study. Exclusion criteria were patients who refused to participate the study or samples that were not technically suitable for nucleic acid extraction.

**RNA extraction and cDNA synthesis: **HIV-1 RNA was isolated from plasma using (Qiagen extraction kit, Düsseldorf, Germany) based on the manufacturer's instructions. The kit yields high quality extracted RNA with minimal contaminations. Next, the cDNA was synthesized using Thermo Scientific RevertAid First Strand cDNA Synthesis Kit (Thermo Fisher Scientific, Waltham, U.S.) based on kit provided manual.

**Nested PCR primers and program: **The primer pairs used for nested PCR to amplify RT gene were based on W.H.O manual for HIV drug resistance assays [21]. These consensus primers have been confirmed for the specificity to HIV-1 RT gene amplification. The primer sequences were as follows: 

F1: 5' TTTYAGRGARCTYAATAARAGAACTCA 3', 

R1: 5' CCTCITTYTTGCATAYTTYCCTGTT 3'; )amplicon size: 846 bp) and

F2: 5' TTYTGGGARGTYCARYTAGGRATACC 3',

R2: 5' GGYTCTTGRTAAATTTGRTATGTCCA 3' )amplicon size: 775 bp) 

In which R represents Guanine/Adenine (purine) and Y corresponds to Cytosine/Thymine (pyrimidine) nucleotides. To set up the optimized condition for DNA polymerase activity the following components in the reaction were used: 10x buffer with MgSO4 (5 µl), dNTP mix (0.2 mM each), primers (1µM each), DNA (~200 ng) and DNA polymerase (2.5 U per reaction). The PCR thermal protocol consisted of initial denaturation step of 95°C for 10 min, followed by 35 cycles of denaturation (95°C for 20 sec), annealing (55°C for 30 sec), extension (72°C for 2.5 min) and a final extension of 72°C for 10 min. The same protocol was used in both nested programs, except that 3 µl DNA from the first PCR product was used in the second round of all nested protocols.

**Gel electrophoresis, gel extraction and sequencing: **The products were then electrophoresed on 1.5% agarose gel. Next, the desired DNA bands were extracted from the gel using a commercial Gel Purification Kit (GenetBio Co, Daejen, South Korea). The yield of the purified product together with sequencing primers were then sent to a molecular company for sequencing (Kawsar Biotech Company,Tehran, Iran). The amplified fragments were bidirectionally sequenced bases on the Sanger method using Applied Biosystems 3730/3730xl DNA Analyzer. The sequences were edited in BioEdit version 7.1.9 using both side readings.

**Data analysis: **Sequencing data were read by Chromas software and the mutations analyzed using HIV Drug Resistance Database (https://hivdb.stanford.edu). The database provides information regarding known HIV drug resistance mutations for each query.

**Ethical considerations: **The study was conducted according to the Helsinki declaration on human research. The project proposal was reviewed and approved by the Ethical Committee of Mashhad University of Medical Sciences (approval code: IR.MUMS.fm.REC.1394.86) and written consent was obtained from all participants. The patient demographic data was provided anonymously from patient's recorded files. 

**Statistical analysis: **To compare age in two groups, independent T test and to compare gender in the study groups chi square tests were used. The presence of mutations in the two groups was compared using Chi square test. The SPSS software version 16.0 was used and a P≤0.05 was considered statistically significant in all analyses.

## Results

Table 1 demonstrates patient’s demographic characteristics. The two groups were age and sex matched (P=0.6 and 1.0 respectively). The most prevalent transmission route was sexual contact. High coinfection with HCV was observed in both groups.

**Table 1:** The demographic data for HIV-1 Positive patients in Mashhad

**Characteristics**	**Total**	**Treatment Naive ** **patients n (%)**	**Under treatment patients** **n (%)**
**Number**	44 (100)	22 (100)	22 (100)
**Mean age**	39.6±7.3	40.8±7.9	38.5±6.7
**Gender**			
**Male**	26 (59)	13 (59)	13 (59)
**Female**	18 (41)	9 (41)	9 (41)
**Transmission rout**			
**Sexually**	24 (54.5)	11 (50)	13 (59)
**IV drug abusers**	16 (36)	9 (41)	7 (32)
**Tattoos history**	1 (2.27)	-	1 (4.5)
**Blood transfusion**	1 (2.27)	1 (4.5)	-
**unknown**	1 (2.27)	-	1 (4.5)
**HCV-association**	18 (41)	9 (41)	9 (41)

The primers' nucleotide blasted for HIV-1 Reverse transcriptase gene and 100% homology was observed using primer BLAST tool. As shown in (Fig. S1), a band with 775 bp corresponding to amplified RT sequences was observed in each sample. GeneRuler 100 bp Plus DNA Ladder, #SM0323, Thermo scientific company, Germany; was used to identify the desired band.

As demonstrated in Table 2, in treatment-naïve patients, several low-level mutations associated with NNRTI minimal drug resistance were observed including E138A, V179T. In addition, high-level resistance mutations to efavirenz (Y188L and Y188F) were detected. No specific mutations to NRTI drugs were observed in these groups, and none of these patients had multiple mutations. In under treatment group, numerous mutations related to NRTI resistance were observed including M184V, T215F, T215Y, K219Q, and L210W. Also, a range of NNRTI resistance mutations was detected as shown in Table 2. These mutations consisted of K101E, K103N, K103E, V106M, V108I, E138A, Y181C, Y188L, Y188H, G190A, and P225H. 

**Table 2:** Drug resistance mutations to NRTIs and NNRTIs among HIV-1 positive patients in Mashhad

**Treatment naive patients (n)**		**Under treatment patients (n)**	
NRTI	NNRTI	NRTI	NNRTI
None	1 (E138A)	9 (M184V)	2 (K101E)
	1 (V179T)	1 (L210W)	1 (K103N)
	1 (Y188L)	3 (T215F)	1 (K103E)
	1 (Y188F)	1 (T215Y)	1 (V106M)
		3 (K219Q)	1 (V108I)
			3 (E138A)
			4 (G190A)
			2 (Y181C)
			3 (Y188L)
			1 (Y188H)
			4 (P225H)

NRTI: nucleoside/nucleotide reverse transcriptase inhibitors. NNRTI: non-nucleoside reverse transcriptase inhibitors

Table 3 demonstrates the level of resistance observed among patients to the antiretroviral drugs. All the above-mentioned mutations are approved by the World Health Organization (WHO) and the International AIDS Society (IAS-USA) listed in https://hivdb.stanford.edu/page/who-sdrm-list [22, 23]. In this study, 45% of under treatment and 9% of treatment-naïve patients carried at least one high-level drug resistance mutation, which was significantly higher in the under treatment group (p<0.01).

**Table 3:** Drug resistance among HIV-1 positve patients in Mashhad (by name and class of drug)

**Drug resistance level in** **Naive treatment patients**				**Drug resistance level in** **Under treatment patients**			
	**HL. ** **n (%)**	**IL. n (%)**	**LL. n (%)**		**HL. ** **n (%)**	**IL. n (%)**	**LL. n (%)**
**NRTI**							
**AZT**	-	-	-	AZT	3(30)	-	3(30)
**3TC**	-	-	-	3TC	6(60)	-	-
**ABC**	-	-	-	ABC	-	-	9(90)
**FTC**	-	-	-	FTC	6(60)	-	-
**TDF**	-	-	-	TDF	-	-	3(30)
		-		ddI	-	-	9(90)
		-		d4T	3(30)	-	-
**NNRTI**		-					
**EFV**	2(9)	-	-	EFV	5(50)	3(30)	1(10)
**ETR**	-	-	2(9)	ETR	-	-	8(80)
**NVP**	2(9)	-	-	NVP	8(80)	-	-
**RPV**	1(4.5)	1(4.5)	1(4.5)	RPV	3(30)	-	4(40)

HL: high level, IL: intermediate level, LL: low level, AZT: Zidovudine, 3TC: Lamivudine, ABC: abacavir, FTC: Emtricitabine, TDF: tenofovir, ddI: Didanosine, d4T: Stavudine, EFV: Efavirenz, ETR: Etravirine, NVP: Nevirapine, RPV: Rilpivirine.

## Discussion

Here we report the drug resistance mutations to RT inhibitor among a group of the patients in Mashhad, northeastern Iran. We have previously reported PI mutations in the region [24]. The pattern of mutations undoubtedly helps physicians to choose the best regimen and to improve national guidelines for HIV treatment.

In our study, NRTI-resistant mutation M184V, which provides high-level resistance to 3TC and FTC, and low-level resistance to ABC and ddI was detected as a circulating mutation among out HIV-1 infected patients. Also, T215F which produces high-level resistance to AZT and d4T and low-level resistance to ddI , TDF and ABC was detected in our patients. In addition, K219Q mutation developing low-level resistance to AZT, and L210W develops low-level resistance to ZDV. T215Y, L210W, and K219Q are non-polymorphic and thymidine analog mutations (TAMs) first seen in patients receiving a single AZT. The most common mutation causing NRTI resistance is M184V/I, which alone produces high-level resistance to both lamivudine and emtricitabine, followed by a series of mutations related to the thymidine analogue (TAMs). Common TAMs are: K219Q/E, T215Y/F, L210W, K70R, D67N and M41L. Accumulation of TAMs leads to resistance to multiple NRTIs [25]. HIV variants containing several TAMs have been observed in patients failing treatment with zidovudine and lamivudine [26].

Regarding NNRTI resistance mutations, the most frequent mutations found in the present study were E138A, G120A, P225H and Y188L. The mutation E138A confers low-level resistance to RPV and ETR, while G190A provides high-level resistance to NVP and medium-level resistance to EFV. P225H is a non-polymorphic mutation that provides high level resistant to EFV, and Y188L is a non-polymorphic resistance mutation that confers high level resistance to NVP/EFV and reduces susceptibility to RPV and ETR [27]. The high level resistance mutations with significant clinical importance in our study were K103N, G120A, Y188L, Y181C, and P225H.

Such studies should be updated due to the emergence of new drug resistance mutations for example in the report of the International AIDS Society in the United States (IAS-USA) in 2013, M230L mutation was added to the list of mutations resistant to two NNRTIs (efavirenz and nevirapine). The study reported K65R and M184V/I as lamivudine-resistant mutations; M41L, D67N, K70R, L210W, T215YF and K219Q/E Zidovudine resistance mutations; and Y188L, G190SA P225H, M230L, L100I, K101P, K103N/S, V106M, V108I, Y181C/I as resistance mutations to efavirenz [28].

Three common NNRTI-resistant mutations have been K103N, Y181C, and G190A, all were observed in our study. One possible reason why the K103N mutation is more common is that efavirenz and nevirapine are widely used in the first line of treatment; thereby K103N is most frequently found in regimens containing efavirenz [29].

Different factors play a critical role in the accumulation of drug resistance mutations, among which treatment regimen has been noticed. A study conducted in 2012 in Switzerland included five first-line compounds (5 drug regimen groups): zidovudine (azt)+ efavirenz (FEV), tenofovir (TDF)+EFV, lopinavir (LPV)+ AZT, LPV+TDF, ritonavir-boosted atazanavir (ATZ / r)+TDF. The study showed that EFV+AZT had the highest prevalence of cumulative resistance, compared to all treatment regimens [30]. Similarly, treatment pressure may affect the circulating mutated strains in different patients, for example, a study reported that the most common NNRTI resistance mutation in the rilpivirine group was E138K, while in the efavirenz group, K103N was the major NNRTI resistance mutation [31]. Also, N348I has been frequently observed in therapies containing AZT and/or ddI and is associated with TAMs such as T215Y [32]. In patients prescribed rilpivirine (RPV) in combination with lamivudine and emtricitabine, E138K and M184I have been frequently reported [33].

The mutation pattern mainly is linked with a national regimen, for example, the main mutations reported against first-line treatment among Ethiopian patients have been K103N and M184V [34]. In this regards, in China, NRTI and NNRTI-related mutations including M41L, T215Y/F, D67N, K103N, G190A/S, Y181C/F and L210W have been observed [35].

In Iran, a study conducted in Tehran showed that among NRTI drugs, lamivudine with a 24% rate had the highest resistance while no resistance to tenofovir was observed. This can be explained mainly due to the high prescription of lamivudine comparing limited tenofovir usage. Almost similar results were obtained for zidovudine and abacavir [36]. In Gorgan north of Iran, in treatment naïve patients, 15% had resistance to NRTIs and 20% showed resistance to NNRTIs and no resistance were observed against protease inhibitors. While, in under treatment group, 25% resistance to NRTIs, 20% resistance to NNRTIs and 5% resistance to protease inhibitors were observed [37]. In another study in Iran, 16 out of 24 patients (66.6%) showed resistance mutations to NRTI while 8 patients (32%) had resistance mutations to NNRTIs. The study showed high-level resistance to 3TC, NVP and EFV [38]. The transfer of resistance to NRTI in Iran has been a main concern since the first line of antiretroviral therapy has been designed based on the combination of NRTI + NNRTI. Transfer of resistance to protease inhibitors is expected to be low, however it should be noted that these drugs are less commonly prescribed in Iran. 

A synergism in mutation effects have been shown, for example, N348I develops resistance to Zidovudine and Nevirapine. This mutation also reduces the susceptibility to nevirapine (7.5-fold) and efavirenz (2.5-fold) and clearly enhances resistance to these drugs when combined with K103N mutation [39]. Similarly, in patients receiving lamivudine and Emtricitabine NRTIs, the M184I/V and E138K mutations have synergetic interaction [40].

Resistance to first-generation NNRTIs is mainly relevant to several codons, but the K103N or Y181C mutation alone induce clinical failure of delavirdine, efavirenz, and nevirapine therapy [41]. NNRTIs have a long plasma half-life that renders long suboptimal remaining of the drug, therefore variable levels of drugs remain in the plasma for more than a few weeks, which speeds up the development of NNRTI resistance mutations. E138A is a mutation selected in patients using two NNRTIs, ETR and RPV and reduces the sensitivity to these drugs. This mutation was observed in two cases in our study. One patient received ETR and RPV medications for a limited time; however, the other patient had no history of ART medication. Similarly, NNRTI-resistant mutations: V179T, Y188L, and Y188F were detected in treatment naïve group which might have been transmitted by resistant virus transmission to these patients. Nationally drug resistance mutations have been reported from different centers [42, 43]. and it seems that upcoming mutations are a great concern. Similar to our results, higher mutation rates have been reported among under treatment cases [38]. which may indicate suboptimal therapy. 

In the present study, resistance mutations to both NRTIs and NNRTIs were observed. Some mutations cause high-level drug resistance and are of great clinical importance which indicates that suboptimal therapy should be prevented to reduce the rate of the circulating mutations. A limitation of the study was a rather small sample size, and large-scale studies may complete the gaps. Also, it is recommendable that drug resistance screening studies be conducted over the years to reveal possible emerging mutations.

## Conflict of Interest:

 The author declared no conflict of interest.

## Authors’ Contribution:

ZM: Performed sampling and experimental work, worte the manuscript with MY; STH: helped in experimental setup; KB: helped in data analysis; SAJ: co-supervised the experimental work; KG: helped in data interpretation and discussion; MY: principal investigator, supervised the research project, worte the manuscript with ZM.

## Supplementary materials

Figure S1 Amplified RT sequences of 5 representative patients. M: marker, NC: negative control.

## References

[1] Haider MR, Gao D, Zou Z, Dong B, Zhang W, Chen T, Cui W, Ma Y (2019). Secular trends in HIV/AIDS mortality in China from 1990 to 2016: Gender disparities. PLos One.

[2] Haghdoost AA, Mostafavi E, Mirzadeh A, Navadeh S (2011). Modeling of HIV/AIDS in Iran up to 2014. J AIDS HIV Res.

[3] Gharagozloo M, Doroudchi M, Farjadian S, Pezeshki AM, Ghaderi A (2005). The frequency of CCR5Δ32 and CCR2-64I in southern Iranian normal population. Immunol Lett.

[4] Perrino T, Gonzalez-Soldevilla A, Pantin H, Szapocznik J (2000). The role of families in adolescent HIV prevention: A review. Clin Child Fam Psychol Rev.

[5] Barbaro G, Scozzafava A, Mastrolorenzo A, Supuran CT (2005). Highly active antiretroviral therapy: current state of the art, new agents and their pharmacological interactions useful for improving therapeutic outcome. Curr Pharm Des.

[6] Margolis AM, Heverling H, Pham PA, Stolbach A (2014). A review of the toxicity of HIV medications. J Med Toxicol.

[7] Hamers RL, de Wit TFR, Holmes CB (2018). HIV drug resistance in low-income and middle-income countries. Lancet HIV.

[8] Rojas Sánchez P, Holguín A (2014). Drug resistance in the HIV-1-infected paediatric population worldwide: a systematic review. J Antimicrob Chemother.

[9] Sluis-Cremer N, Arion D, Parniak M (2000). Molecular mechanisms of HIV-1 resistance to nucleoside reverse transcriptase inhibitors (NRTIs). Cell Mol Life Sci.

[10] Holec AD, Mandal S, Prathipati PK, Destache CJ (2017). Nucleotide reverse transcriptase inhibitors: a thorough review, present status and future perspective as HIV therapeutics. Curr HIV Res.

[11] de Béthune MP (2010). Non-nucleoside reverse transcriptase inhibitors (NNRTIs), their discovery, development, and use in the treatment of HIV-1 infection: a review of the last 20 years (1989–2009). Antiviral Res.

[12] Zapor MJ, Cozza KL, Wynn GH, Wortmann GW, Armstrong SC (2004). Antiretrovirals, Part II: focus on non-protease inhibitor antiretrovirals (NRTIs, NNRTIs, and fusion inhibitors). Psychosomatics.

[13] Sierra-Aragon S, Walter H (2012). Targets for inhibition of HIV replication: entry, enzyme action, release and maturation. Intervirology.

[14] D’Aquila RT, Schapiro JM, Brun-Vézinet F, Clotet B, Conway B, Demeter LM, Grant RM, Johnson VA, Kuritzkes DR, Loveday C, Shafer RW, Richman DD (2003). Drug resistance mutations in HIV-1. Top HIV Med.

[15] De Clercq E (2009). Anti-HIV drugs: 25 compounds approved within 25 years after the discovery of HIV. Int J Antimicrob Agents.

[16] Weller IV, Williams I (2001). ABC of AIDS. Antiretroviral drugs. BMJ.

[17] Sluis-Cremer N, Temiz NA, Bahar I (2004). Conformational changes in HIV-1 reverse transcriptase induced by nonnucleoside reverse transcriptase inhibitor binding. Curr HIV Res.

[18] Mirzaei M, Farhadian M, Poorolajal J, Afasr Kazerooni P, Tayeri K, Mohammadi Y (2018). Life expectancy of HIV-positive patients after diagnosis in Iran from 1986 to 2016: A retrospective cohort study at national and sub-national levels. Epidemiol Health.

[19] Bagheri Amiri F, Doosti-Irani A, Sedaghat A, Fahimfar N, Mostafavi E (2017). Knowledge, Attitude, and Practices Regarding HIV and TB Among Homeless People in Tehran, Iran. Int J Health Policy Manag.

[20] Secretariat NAC (2015). Islamic Republic of Iran AIDS Progress Report.

[21] Edition T, Licence: CC BY-NC-SA 3.0 IGO (2020). WHO manual for HIV drug resistance testing using dried blood spot specimens.

[22] Bennett DE, Camacho RJ, Otelea D, Kuritzkes DR, Fleury H, Kiuchi M, Heneine W, Kantor R, Jordan MR, Schapiro JM, Vandamme AM, Sandstrom P, Boucher CAB, Van de Vijver D, Rhee SY, Liu TF, Pillay D, Shafer RW (2009). Drug resistance mutations for surveillance of transmitted HIV-1 drug-resistance: 2009 update. PLoS One.

[23] Shafer RW, Rhee SY, Pillay D, Miller V, Sandstrom P, Schapiro JM, Kuritzkes DR, Bennett D (2007). HIV-1 protease and reverse transcriptase mutations for drug resistance surveillance. AIDS.

[24] Nasiri-Tajabadi Z, Salim FB, Najafzadeh MJ, Kalantari S, Garshasbi S, Jamehdar SA, Farsiani H, Mazaheri Z, Sankian M, Youssefi M (2018). A surveillance on protease inhibitor resistance-associated mutations among Iranian HIV-1 patients. Arch Clin Infect Dis.

[25] Hu Z, Giguel F, Hatano H, Reid P, Lu J, Kuritzkes DR (2006). Fitness comparison of thymidine analog resistance pathways in human immunodeficiency virus type 1. J Virol.

[26] Betancor G, Nevot M, Mendieta J, Gómez-Puertas P, Martínez MA, Menéndez-Arias L (2014). Molecular basis of the association of H208Y and thymidine analogue resistance mutations M41L, L210W and T215Y in the HIV-1 reverse transcriptase of treated patients. Antiviral Res.

[27] Clavel F, Hance AJ (2004). HIV drug resistance. N Engl J Med.

[28] Johnson VA, Calvez V, Günthard HF, Paredes R, Pillay D, Shafer RW, Wensing AM, Richman DD (2013). Update of the drug resistance mutations in HIV-1: March 2013. Top Antivir Med.

[29] Ibe S, Sugiura W (2011). Clinical significance of HIV reverse-transcriptase inhibitor-resistance mutations. Future Microbiol.

[30] von Wyl V, Yerly S, Böni J, Shah C, Cellerai C, Klimkait T, Battegay M, Bernasconi E, Cavassini M, Furrer H, Hirschel B, Vernazza PL, Ledergerber B, Gunthard HF, Swiss HIV Cohort Study (2012). Incidence of HIV-1 Drug Resistance Among Antiretroviral Treatment–Naive Individuals Starting Modern Therapy Combinations. Clin Infect Dis.

[31] Molina JM, Cahn P, Grinsztejn B, Lazzarin A, Mills A, Saag M, Supparatpinyo K, Walmsley S, Crauwels H, Rimsky LT, Vanveggel S, Boven K, ECHO study group (2011). Rilpivirine versus efavirenz with tenofovir and emtricitabine in treatment-naive adults infected with HIV-1 (ECHO): a phase 3 randomised double-blind active-controlled trial. Lancet.

[32] Hachiya A, Kodama EN, Sarafianos SG, Schuckmann MM, Sakagami Y, Matsuoka M, Takiguchi M, Gatanaga H, Oka S (2008). Amino acid mutation N348I in the connection subdomain of human immunodeficiency virus type 1 reverse transcriptase confers multiclass resistance to nucleoside and nonnucleoside reverse transcriptase inhibitors. J Virol.

[33] Hu Z, Kuritzkes DR (2011). Interaction of reverse transcriptase (RT) mutations conferring resistance to lamivudine and etravirine: effects on fitness and RT activity of human immunodeficiency virus type 1. J Virol.

[34] Abdissa A, Yilma D, Fonager J, Audelin AM, Christensen LH, Olsen MF, Tesfaye M, Kaestel P, Girma T, Aseffa A, Friis H, Pedersen C, Andersen AB (2014). Drug resistance in HIV patients with virological failure or slow virological response to antiretroviral therapy in Ethiopia. BMC Infect Dis.

[35] Gong J, Wang XQ, Tong X, Shen XH, Yang RG (2011). Emerging trends of drug-resistant HIV-1 among drug-treated patients in former blood donors in Hubei, China: a three-year surveillance from 2004 to 2006. Virol Sin.

[36] Hajabdulbaghy M, Soodbakhsh A, Soleimani A (2011). The prevalence of drug resistance in patients with HIV/AIDS attending to Imam Khomeini Hospital in Tehran, Iran during 2008-2009: letter to editor. Tehran University Med J.

[37] Naziri H, Tabarraei A, Ghaemi A, Davarpanah MA, Javid N, Moradi A (2013). Drug-Resistance-Associated Mutations and HIV Sub-Type Determination in Drug-Naïve and HIV-Positive Patients under Treatment with Antiretroviral Drugs. Med Lab J.

[38] Baesi K, Ravanshad M, Hosseini Y, Haji Abdolbaghi M (2012). Drug resistance profile and subtyping of HIV-1 RT gene among Iranian under-treatment patients. Iran J Biotechnol.

[39] Yap SH, Sheen CW, Fahey J, Zanin M, Tyssen D, Lima VD, Wynhoven B, Kuiper M, Sluis-Cremer N, Harrigan PR, Tachedjian G (2007). N348I in the connection domain of HIV-1 reverse transcriptase confers zidovudine and nevirapine resistance. PLoS Med.

[40] Xu HT, Oliveira M, Quashie PK, McCallum M, Han Y, Quan Y, Brenner BG, Wainberg MA (2012). Subunit-selective mutational analysis and tissue culture evaluations of the interactions of the E138K and M184I mutations in HIV-1 reverse transcriptase. J Virol.

[41] Johnson VA, Brun-Vézinet F, Clotet B, Gunthard HF, Kuritzkes DR, Pillay D, Schapiro JM, Richman DD (2007). Update of the drug resistance mutations in HIV-1: 2007. Top HIV Med.

[42] Baesi K, Ravanshad M, Ghanbarisafari M, Saberfar E, Seyedalinaghi S, Volk JE (2014). Antiretroviral drug resistance among antiretroviral‐naïve and treatment experienced patients infected with HIV in Iran. J Med Virol.

[43] Naziri H, Baesi K, Moradi A, Aghasadeghi MR, Tabarraei A, McFarland W, Davarpanah MA (2016). Antiretroviral drug resistance mutations in naïve and experienced patients in Shiraz, Iran, 2014. Arch Virol.

