# Supplementary material for: A survey of resistance mutations to reverse transcriptase inhibitors (RTIs) among HIV-1 patients in northeast of Iran
Source: Mol Biol Res Commun. 2024;13(3):117–25. doi: 10.22099/mbrc.2024.48729.1895 (PMC11194027; doi:10.22099/mbrc.2024.48729.1895)
Supplement: Figure S1 [file mbrc-13-117-s001.pdf]

A survey of resistance mutations to reverse transcriptase inhibitors (RTIs) among HIV-1 patients in northeast of Iran

Zahra Mazaheri[1], Sahar Tahaghoghi-Hajghorbani[2], Kazem Baesi[3], Kiarash Ghazvini[1,4], Saeid Amel-Jamehdar[1,4], Masoud Youssefi[1,4,*]

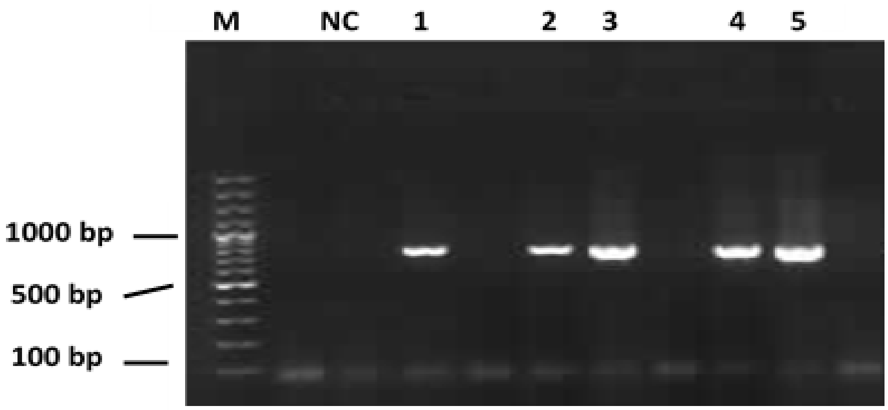

Figure S1: Amplified RT sequences of 5 representative patients. M: marker, NC: negative control.
